# Polymer-Based Functional Materials Loaded with Metal-Based Nanoparticles as Potential Scaffolds for the Management of Infected Wounds

**DOI:** 10.3390/pharmaceutics16020155

**Published:** 2024-01-23

**Authors:** Xhamla Nqoro, Raymond Taziwa

**Affiliations:** Department of Applied Science, Faculty of Natural Sciences, Walter Sisulu University, Old King William’s Town Road, Potsdam Site, East London 5200, South Africa; rtaziwa@wsu.ac.za

**Keywords:** wound infection, antibacterial, metal nanoparticles, polymeric dressings

## Abstract

Wound infection due to bacterial invasion at the wound site is one of the primary challenges associated with delayed wound healing. Microorganisms tend to form biofilms that protect them from harm, leading to their multidrug resistance. The alarming increase in antibiotic resistance poses a threat to wound healing. Hence, the urgent need for novel wound dressing materials capable of managing bacterial infection is crucial for expediting wound recovery. There is considerable interest in polymeric wound dressings embedded with bioactive substances, such as metal-based nanoparticles, as potential solutions for treating microbially infected wounds. Metal-based nanoparticles have been widely used for the management of infected wounds due to their broad antimicrobial efficacy. This review focuses on polymer-based and bioactive wound dressings loaded with metal-based nanoparticles like silver, gold, magnesium oxide, or zinc oxide. When compared, zinc oxide-loaded dressings exhibited higher antibacterial activity against Gram-positive strains and silver nanoparticle-loaded dressings against gram-negative strains. However, wound dressings infused with both nanoparticles displayed a synergistic effect against both strains of bacteria. Furthermore, these dressings displayed antibiofilm activity and the generation of reactive oxygen species while accelerating wound closure both in vitro and in vivo.

## 1. Introduction

Skin is the body’s first line of defense against the external environment. It is specialized with protective immune cells that help to orchestrate tissue repair and fight infection [1].Wound healing is an intrinsic mechanism that occurs after skin injury, and it is achieved through complex overlapping phases, namely haemostasis, inflammation, proliferation, and maturation [2,3]. The inflammation phase is the one that is most targeted by bacterial infection, and it is accompanied by wound exudate, persistent inflammation, and severe pain [4]. Bacterial organisms agglomerate at the wound surface and form a biofilm that protects them against harm from bodily cells and external treatment [5]. At this stage, microorganisms and various other limiting factors such as immunodeficiency, metabolic conditions, ischemic conditions, growth factor activity, etc. hinder the progression of the inflammatory phase to the proliferation phase by producing inflammatory mediators, resulting in an overproduction of reactive oxygen species [6]. The significant imbalance between bacterial proliferation and the host’s response results in heightened local inflammation and a prolonged chronic inflammation phase. This leads to cell death. Moreover, due to this, microorganisms have developed multidrug resistance, posing an alarming challenge in antibiotic treatment [7]. Improper treatment for chronic infections may result in an increased risk of amputation [8,9]. In recent times, different approaches have been developed for the treatment of infected wounds. Metal-based nanoparticles have shown a great potential due to their intrinsic antibacterial properties.

**Figure 1:**
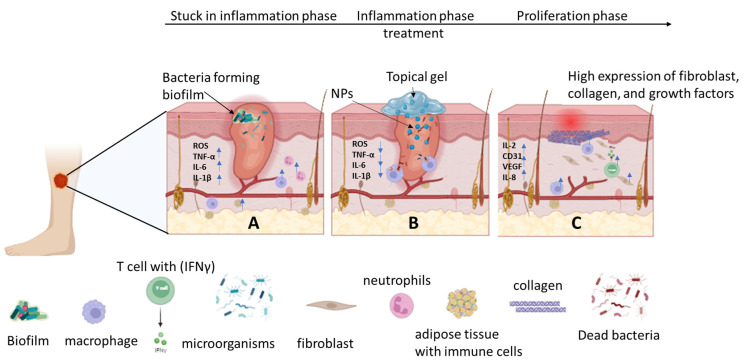
A: Illustrates a wound stuck in the inflammation phase due to bacterial infection, showcasing elevated levels of inflammatory factors. B: Depicts the treatment of the wound with a topical gel loaded with nanoparticles resulting in reduced levels of inflammatory factors, ROS, and bacterial death. C: Shows the wound progressing to the proliferation phase post-treatment, exhibiting increased levels of growth factors, immune cells, endothelial cells, and collagen expression. The blue arrows pointing up indicate increase of the growth factors, immune cells and ROS, while blue arrows pointing down indicate decrease of inflammatory cells and ROS.

Biomedical applications of metal-based nanoparticles have gained a lot of attention due to their unique features such as antibacterial efficacy, magnetism, nano-size, optical properties, large surface-area-to-volume ratio, ability to destroy cell walls, and ability to promote the bio-separation of DNA and proteins [10,11]. These features are key to inhibiting bacterial growth and penetrating biofilms. Nevertheless, the biocompatibility of these materials with human skin cells should not be overlooked. Their cytotoxic side effects are linked to their concentration, generation of reactive oxygen species (ROS), instigation of irritation, size, crystalline structure, shape, time of exposure, source of extraction, and other relevant factors [12,13,14]. Various strategies are practiced to overcome these limitations, including their fabrication into polymeric wound dressings. Polymeric wound dressings are ideal scaffolds for wound management due to their suitability for controlled drug delivery, non-toxicity, ease of recognition by human cells, wound exudate absorption capacity, water-vapor transmission rate, antimicrobial properties, and more [15,16]. Figure 1 below shows the wound-healing effects of polymer-based wound dressings loaded with metal-based nanoparticles. This review outlines the wound-healing effects of polymer-based dressings loaded with metal-based nanoparticles for the treatment of infected or chronic wounds.

## 2. Factors That Contribute to Delayed Wound Healing

**Figure 2:**
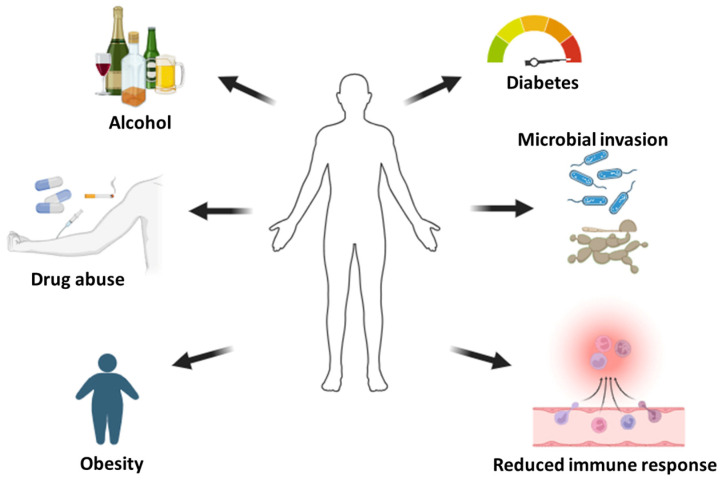
Factors that contribute to delayed wound healing.

In Figure 2, several factors contribute to the impairment of the wound healing process. These factors encompass immune system deficiencies, metabolic conditions, and infections, ultimately contributing to the development of chronic wounds.

### 2.1. Immune System Deficiency

When the body sustains an injury, the immune system responds by employing diverse cell signaling pathways to trigger the production of several repair mechanisms to protect and heal the body [17]. The initial responders to the wound site are neutrophils, functioning as the primary defense against pathogen invasion by releasing antimicrobial peptides, reactive oxygen species, and antimicrobial proteases [18]. The neutrophils also secrete growth factors and cytokines that stimulate the proliferation of endothelial cells, keratinocytes, and fibroblasts [18]. Other immune cells involved in the adaptive response include Langerhans cells, mast cells, T lymphocytes (T cells), and macrophages [1]. Macrophages are classically activated by Interferon gamma signals to M1 macrophages, which are “pro-inflammatory”. The macrophages are switched by AMP-activated protein kinase 1 (AMPKα1) to M2 macrophages, which are “anti-inflammatory” phenotypes and operate throughout the process of progressive wound healing [18,19]. The M1 macrophages express cytokines such as IL1β and tumor necrosis factor (TNFα), and engage in phagocytosis of cellular debris [19,20]. M2 macrophages promote myotubule formation, fusion, and myoblast differentiation, and produce growth factors like vascular endothelial growth factor, fibroblast growth factor, platelet-derived growth factor (PDGF), and transforming growth factor (TGFβ). These cells also express anti-inflammatory cytokines such as IL10 [19,20]. Dysregulation of these pathways may lead to delayed wound healing and chronic wound formation [21]. Interferon gamma (IFN-γ) chemokines secreted by macrophages recruit T lymphocytes (T cells) to the wound site, thereby facilitating the progression of the inflammation phase [22]. The presence of T cells at the wound site is associated with delayed wound healing [23]. Wang et al. compared the wound healing of mice lacking T cells against that of wild-type healthy mice and discovered a fast wound closure in the mice lacking T cells [24]. However, this closure was accompanied by excessive inflammation, scar formation, and abnormal healing compared to the wild-type mice. There are contradictory outcomes on the importance of T cells for progressive wound healing. Various other types of innate lymphocytes, such as γ/δ T cells, natural killer cells, etc., contribute to the wound healing mechanism [19]. Therefore, it is important to develop wound dressing materials that will potentially activate immune responses, especially in patients with immune system deficiency disorders.

### 2.2. Metabolic Conditions

Among various metabolic conditions, diabetes mellitus (DM) is one of the major conditions associated with delayed wound healing. Individuals with DM often experience a compromised immune system response and an imbalance in extracellular matrix regulation, rendering them susceptible to chronic diabetic ulcers [25,26]. DM reduces skin elasticity, delaying dermis regeneration and reducing the feeling of systematic pain, thus leading to delayed wound healing and chronic wound formation [27]. Increased blood glucose levels (hyperglycaemia) result in inadequate blood circulation, reduced oxygenation, and microvascular dysfunction, restricting the amount of nutrients and growth factors delivered to the wound site and systematically leading to persistent inflammation and delayed wound healing [28,29,30]. Furthermore, hyperglycaemia alters the function of the electron transport chain, leading to inadequate adenosine triphosphate (ATP), the excessive release of a great deal of mitochondrial ROS, and microvascular damage [31]. Additionally, hyperglycaemia hinders the migration of crucial leukocytes necessary for combating infection [25], increases overexpression of matrix metalloproteinases (MMPs), promotes biofilm formation and bacterial colonization, and impairs granulation tissue formation [32]. Persistent inflammatory macrophages at the wound site contribute to heightened ROS and pro-inflammatory cytokines expression, leading to decreased angiogenic response, abnormal apoptosis of keratinocytes and fibroblasts, and tissue necrosis [26,33]. Non-healing wounds pose a significant risk of uncontrolled bacterial growth and may ultimately necessitate amputation.

### 2.3. Infection

Microorganisms are one of the major challenges associated with delayed wound healing, resulting from their resistance to multiple drug scaffolds used in wound management. There are various types of bacterial microorganisms that are classified as gram-negative and Gram-positive. Pseudomonas aeruginosa is a gram-negative strain of bacteria most commonly found in chronic and burn wounds [34,35]. This bacterial pathogen delays wound healing by weakening mechanisms associated with the repair of epithelial tissue and damaging epithelial cells [36]. Pseudomonas aeruginosa is resistant to a vast number of drugs used in wound healing, including antibiotics [37]. The resistance of *P. aeruginosa* is linked to its ability to form a biofilm and genetic mutations that allow it to adapt to different environments [36,37,38]. *Staphylococcus aureus* is a common strain of Gram-positive bacteria responsible for various wound infections. It is commonly found in a chronic wound’s top layer, forming a biofilm [39]. *B. subtilis* is a Gram-positive pathogen that forms endospores that enable it to survive harsh conditions such as alkaline, acidic, osmotic, and nutrition-lacking environments [40].

*Staphylococcus aureus* delays wound healing by producing virulent factors such as coagulase, α-toxin, metalloprotease, and enterotoxins that destroy cell membranes, resulting in cell death [41]. Moreover, molecules covering its cell membrane, such as lipoprotein and peptidoglycan, induce an enhanced pro-inflammatory response by activating immune cells through pathogen recognition receptors [41]. The mechanism of resistance for *S. aureus* to various antibiotics includes its β-lactamase enzymes, which are responsible for breaking the β-lactam antibiotic’s ring [42]. *E. faecalis* is commonly found in diabetic and burn wounds, delays re-epithelialization, and has developed resistance due to its acquired and intrinsic resistance to a range of antibiotics [40]. Overall, bacterial infection alters the expression of pro-inflammatory cytokines, increases lactate deposition, disrupts pH levels, elevates the levels of ROS production, and fosters the development of proteases and toxins [5]. These alterations contribute to the prolonged presence of neutrophils and macrophages, trapping the wound in a self-perpetuating inflammation cycle [5]. The prolonged inflammation cycle leads to overproduction of MMPs, a family of proteases responsible for degrading the extracellular matrix [43].

## 3. Metal-Based Nanoparticles in Infected Wound Healing

**Figure 3:**
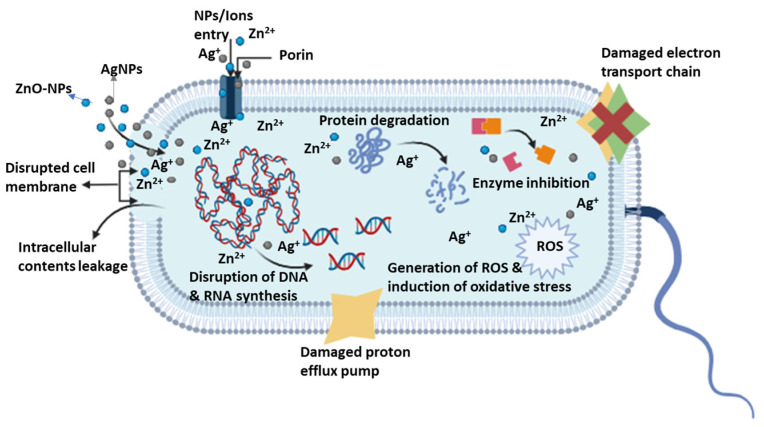
The antibacterial mechanism of zinc oxide and silver nanoparticles.

Antibacterial efficacy is a pre-requisite to progressive wound healing. Metal-based nanoparticles demonstrate higher antibacterial stability than various organic antibacterial molecules, which are often countered by bacterial resistance [44,45]. The development of resistance to metal-based nanoparticles by pathogens is rare, due to the short exposure to nanoparticle radicals as compared to organic radicals. Metal-based nanoparticles, due to their size, surface area, charge, generation of ROS, and morphology, possess high bacterial and biofilm penetration capacity, which antagonizes bacterial growth [46,47,48]. However, higher doses, concentrations, or sizes of these nanoparticles may result in cell toxicity [14]. The cytotoxic effect of metal-based nanoparticles is linked to their interaction with eukaryotic cells. It causes apoptosis, DNA damage, and overproduction of ROS, which affects the respiratory chain of mitochondria and leads to cell damage [49]. The dose and concentration of the nanoparticles is often controlled during the synthesis of the wound dressings and managed via sustained release from the wound dressings into which they are incorporated. Rata et al. noted a decreased cell toxicity of zinc oxide nanoparticles when they were loaded into polymer-based wound dressings, and that their cytotoxic effect is concentration- or dose-dependent [50]. Figure 3 below provides a schematic representation illustrating how nanoparticles inhibit bacterial growth and induce cell death.

### 3.1. Zinc Oxide Nanoparticles (ZnO-NPs)

Zinc is readily available throughout the body and is associated with the regulation of the immune cells that fight off bacterial invasion. It also promotes the production of inflammatory cytokines by inhibiting T cell receptor proteins. Zinc oxide is a hydrated nanoform of zinc, and its derivatives are widely used in the management of infected wounds due to their ability to produce ROS and trigger growth factor-mediated pathways, thus promoting wound healing [51,52]. The antibacterial activity of ZnO-NPs relies on their ability to disrupt the microbial cell wall [53]. Upon breakdown, ZnO-NPs release positively charged Zn^2+^ ions, enhancing interaction with the negatively charged bacterial cell membrane, where they penetrate and rupture the intracellular matrix [13]. The Zn^2+^ ions, upon penetrating the bacterial cell membrane, quickly get hydrated into ZnO and generate ROS, which interact with intracellular fluids, leading to cell death [54,55,56,57]. Li et al. reported ZnO-NP-loaded hydrogels demonstrated high antibacterial efficacy and noted that hydrogels without ZnO exhibited minimal antibacterial activity [58]. Zinc oxide induces bacterial cell death by accumulating inside the cell and disrupting DNA replication and ATP synthesis. This affects mitochondrial function and thus destabilizes the cell membrane, causing membrane rupture and intracellular leakage [58]. This process also exposes and makes the bacteria vulnerable to phagocytic cells, leading to their elimination by macrophages [55]. Smaller zinc oxide nanoparticles (ZnO-NPs) exhibit a heightened antimicrobial effect owing to their high surface-to-volume ratio. This characteristic enhances the contact area between the nanoparticles and the bacterial surface, rendering the microbes more susceptible to the activity of ZnO-NPs [57,59].

### 3.2. Silver Nanoparticles (AgNPs)

The application of AgNPs for the healing of chronic wounds has been widely practiced due to various advantages, such as speeding up wound healing and controlling bacterial infection. Singh et al. stated that AgNPs fostered rapid wound reduction without scarring by controlling the release of anti-inflammatory cytokines, differentiating myofibroblasts from normal fibroblasts, and stimulating keratinocyte proliferation and migration [57]. Palanisamy et al. conducted studies with HaCaT cells treated with AgNPs and noted that at a concentration of 50 µg/mL, these nanoparticles promoted the upregulation of vascular endothelial growth factors and platelet-derived growth factors, crucial in wound healing processes [60]. The Ag^+^ and AgNPs interact with the negatively charged bacterial cell wall, causing membrane damage and leading to the leakage of intracellular contents. They also penetrate the metabolic system of the microbes [61]. Their mechanism of action also involves interactions with phosphorus and sulfur groups of the protein plasma membrane and microbe cell wall, and thus inactivating DNA synthase and respiratory enzymes, resulting in cell death [7,62]. Upon intracellular accumulation of Ag^+^ and AgNPs, the particles interact with bacterial fluids and produce ROS that induce oxidative stress, leading to cell death [63]. This oxidative stress inhibits microbial RNA and DNA replication by impeding ATP synthesis, hindering cell division and growth [13,61]. Additionally, Ag^+^ and AgNPs inhibit enzyme synthesis, denature the microbe’s protein mechanisms, and impair ion exchange [56,64].

### 3.3. Gold Nanoparticles (Au-NPs)

Gold nanoparticles (Au-NPs) have found extensive use in biomedical applications due to their multifaceted properties, including antimicrobial activity, tissue adhesion, antioxidant capabilities, and their effectiveness in laser-activated wound healing [51,65,66]. In wound healing, Au-NPs exhibit various mechanisms of action. They facilitate wound healing by quenching and deactivating ROS such as nitric oxide (NO) and hydrogen peroxide (H_2_O_2_) [13]. Furthermore, they enhance the activation of antioxidant genes (such as NRF2) and stimulate collagen expression, cytokine production, and vascular endothelial growth factors, all pivotal in promoting wound healing processes [13]. The anti-angiogenic and anti-inflammatory effects of Au-NPs contribute to the release of essential proteins necessary for wound healing [61]. Additionally, they enhance the adhesion properties of human fibroblast cells, further aiding in wound closure [67]. Gold nanoparticles also exert their wound healing effects by inhibiting bacterial growth, the transmembrane pumps responsible for drug efflux, and the concentration of expressed β-lactamase, ultimately leading to bacterial cell death [9]. When Au-NPs are taken up by bacteria, they hinder the synthesis of ATP, leading to metabolism collapse [7]. Another mechanism includes the inhibition of ROS-forming enzymes within bacteria, serving as an antioxidant, and they may also inhibit DNA from uncoiling and replicating, leading to accelerated wound healing [53]. Studies employing hydrogels loaded with Au-NPs have demonstrated high efficacy against *E. coli* and *S. aureus* bacteria, effectively promoting rapid wound closure in *S. aureus*-infected wounds (6 mm in diameter) in mice within just 7 days of treatment [68].

### 3.4. Magnesium Oxide Nanoparticles (MgO-NPs)

Due to their biocompatibility, stability, and antimicrobial properties, magnesium oxide nanoparticles (MgO-NPs) find applications in diverse research fields, such as the food industry, water treatment, and wound healing [56,69,70,71]. In the context of wound healing, studies by Liu et al. demonstrated that MgO-incorporated membranes facilitate wound healing in Sprague-Dawley infected wounds [72]. These membranes stimulate the upregulation of pro-healing cytokines, collagen production, fibroblast proliferation, and endothelial cell generation, and reduce the expression of the inflammatory cytokine interleukin 1-alpha (IL-1α) [72]. The nanoparticles also induce wound healing through bacterial inhibition. Their antibacterial mechanism includes the possible generation of ROS that are toxic to the bacteria [73,74]. However, it is not clear whether MgO-NPs inhibit bacteria by producing ROS or whether the bacteria’s superoxide dismutase (SOD) completely neutralizes the oxidative stress [73]. It has been also reported that via their positively charged ions, these particles interact with and damage the bacterial cell membrane, leading to cell death [74]. The interaction causes leakage of intracellular proteins, minerals, and genetic material, potentially resulting in cell death [75,76]. Additionally, Wasfi et al. reported that MgO-NPs impact the generation of the extracellular matrix, a pivotal component of biofilms that shield bacteria from oxidative stress [77]. These nanoparticles also promote the anti-adhesion of bacteria at the wound site, hindering bacterial invasion [76].

## 4. Polymer-Based Wound Dressings Loaded with Bioactive Materials

**Figure 4:**
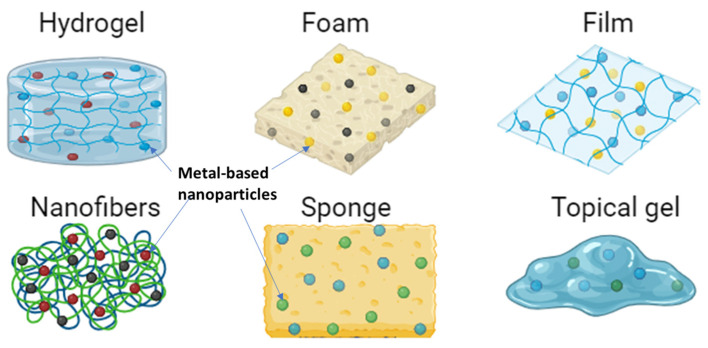
Bioactive dressings loaded with metal-based nanoparticles.

The wound dressing materials reported in this section are classified as bioactive dressings (Figure 4). Bioactive wound dressings are characterized by their ability to deliver bioactive materials such as antibiotics, nanoparticles, growth factors, stem cells, essential oils, vitamins, etc., and these dressings can be functionalized into hydrogels, films, sponges, nanofibers, gels, foams, etc. [16]. These bioactive dressings are synthesized using either synthetic or natural polymers or by hybridizing both to produce a dressing material with the desired wound healing properties.

### 4.1. Bioactive Dressings Loaded with Zinc Oxide Nanoparticles

**Figure 5:**
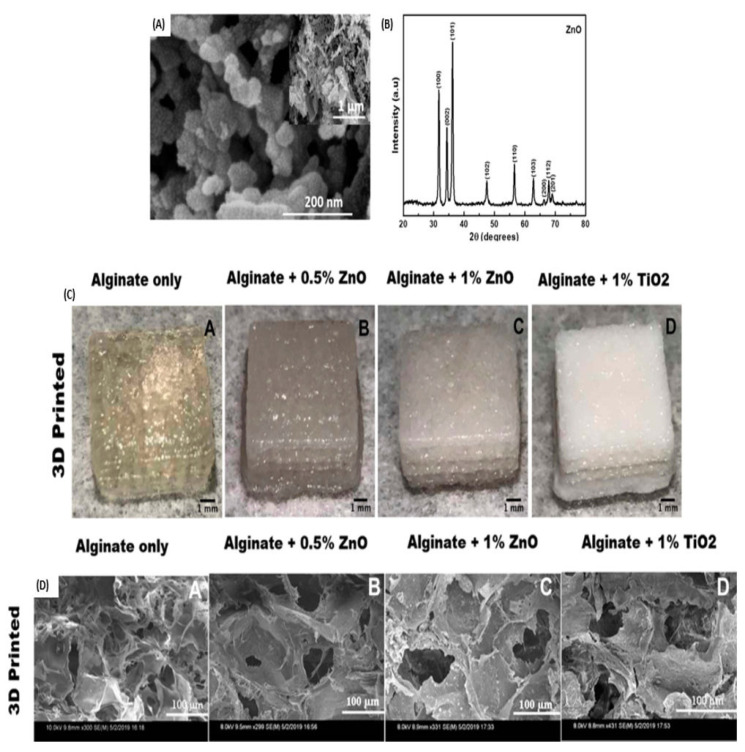
(**A**) SEM image of ZnO-NPs. (**B**) XRD graph of ZnO-NPs. (**C**) 3D printed hydrogels. (**D**) SEM images of 3D printed hydrogels containing different concentrations of ZnO-NPs. (Adapted with permissions from Dove Express [78]. Copyright link https://creativecommons.org/licenses/by-nc/3.0/ (accessed on 23 October 2023).

The application of bioactive dressings for the treatment of chronic wounds is a promising approach due to the features as mentioned above. Their wound-healing features are promoted by the synergistic interactions between the polymers and the nanoparticles. Cleetus et al. developed 3D-printed alginate-based hydrogels loaded with zinc oxide nanoparticles (ZnO-NPs) as potential scaffolds for the treatment of chronic wounds [78]. The reported ZnO-NPs revealed a spherical-shaped morphology with a particle size distribution of 4–6 nm (Figure 5A). Two formulations consisting of 0.5% and 1% ZnO-NPs were synthesized and reported to positively increase the pore size, swelling behavior, and biodegradability of the hydrogels. The 3D-printed hydrogels further displayed high cell viability of mitomycin-C treated STO fibroblasts after treatment. Furthermore, these scaffolds inhibited bacterial growth of S. epidermidis when tested in vitro. The 3D hydrogels reported in this article are potential scaffolds for the promotion of chronic wound healing. However, more studies at a physiological pH that mimics that of infected wounds are recommended. Additionally, tests of in vitro antibacterial efficacy against *S. aureus*, *P. aeruginosa*, and *E. coli* need to be conducted, as these are the main pathogens most often found in chronic wounds.

Shu and co-workers reported chitosan-based biocomposites incorporated with ZnO quantum dots as potential materials for aiding the healing of infected wounds [79]. The reported chitosan-ZnO quantum dots (CS-ZnO QDs) exhibited antioxidant activity against DPPH and ABTS radical scavenging. that is directly proportional to an increase in their dose concentration These biocomposites further displayed antibacterial efficacy against methicillin-resistant *Staphylococcus aureus* (MRSA), showing an 18.7 mm zone of inhibition prepared following the disk agar diffusion method. The anti-biofilm effect of SC-ZnO QDs was recorded 24 and 48 h post-exposure and was noted to decrease from 75.9% to 68.5% as the time of exposure increased. These findings suggest that long-term application of the biocomposite to the biofilm-infected wounds may result in resistance. Alternatively, the decrease could be linked to rapid ZnO QDs release by the chitosan biocomposites. The in vitro cytotoxicity results revealed that even at higher concentrations, the CS-ZnO QDs had no cytotoxic effect on NIH/3T3 fibroblast cells. Their wound healing potential was evaluated in vivo on MRSA-infected BALB/c mice. Wound closure was recorded in 14 days, and after 7 days a 50% wound closure was observed in those treated with SC-ZnO QDs. Those treated with plain CS biocomposite exhibited only 30% wound closure. A 90% wound closure was observed at day 14 post-treatment with SC-ZnO QDs, followed by 87% and 69% after treatment with ZnO and CS biocomposite, respectively. The overall results expressed by these composites indicates a synergistic effect between chitosan and ZnO QDs to promote rapid wound healing.

Rashedi and co-workers developed polylactic acid-based nanofibrous nanocomposites loaded with ZnO and tranexamic acid (PLA/ZnO/TA) and reported their wound healing effects [80]. The particle size of the ZnO-NPs was reported to be 45 nm, with a spherical surface morphology. Three formulations were prepared with varying concentrations of ZnO-NPs. Their antibacterial efficacy against *E. coli* and *S. aureus* was concentration-dependent. PLA/ZnO/TA with 0.5% ZnO exhibited minimal antibacterial efficacy, compared to PLA/ZnO/TA with 2% ZnO against both strains of bacteria. It should be noted that the PLA/ZnO/TA nanofibers were more potent against *S. aureus* than they were against *E. coli*. The nanofibers presented no notable cytotoxicity against mesenchymal stem cells and human skin fibroblast cells after three days of incubation. Additionally, a 90% in vivo wound closure in mice was reported after 14 days post-treatment compared to the control mice, which showed only a 65% wound closure. The H&E staining of PLA/ZnO/TA-treated groups showed complete deposition of collagen and re-epithelialization, compared to the control groups, which showed random depositions of collagen bundles.

**Figure 6:**
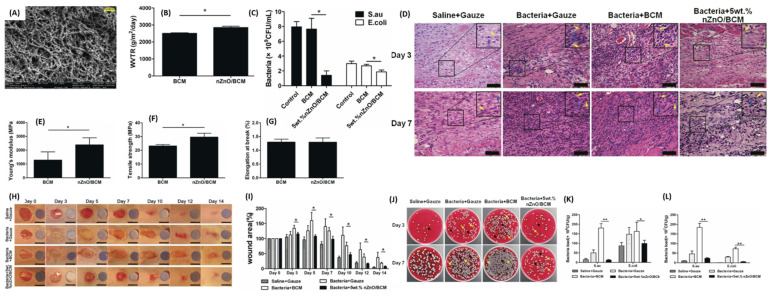
(**A**) SEM image of nanofibers fabricated with 5 wt.% ZnO. (**B**) WVTR of the nanofibers. (**C**) in vitro antibacterial activity of different dressings after 24 h of incubation (* *p* < 0.05, mean ± SEM *n* = 4). (**D**) H&E staining images of groups treated with different dressings after 3- and 7-days post-injury; yellow arrows represent indicate inflammatory cells and blue arrows indicate fibroblasts. Mechanical properties of the prepared dressings (**E**) Young’s modulus, (**F**) Tensile strength, (**G**) Elongation at break point. In vivo wound closure (**H**) and quantitative wound area (**I**) after treatment with different dressings. (**J**) Photographs and (**K**,**L**) quantitative counts of bacterial colonies formed by *S. aureus* and *E. coli* obtained from wound tissues. The yellow arrows represent the colony of *E. coli*; the black arrows indicate the colony of *S. aureus*. (* *p* < 0.05, ** *p* < 0.01, mean ± SEM *n* = 4). Adapted with permission from Dove Express [81]. CC-BY copyright license.

Luo et al. fabricated ZnO-NPs in a bacterial cellulose-based membrane (ZnO/BCM) and confirmed their cell safety using an MTT assay on mammalian cells [81]. These membranes were comfortable, flexible, and strong, showing their suitability for application in wound healing (Figure 6B–E). In vitro antimicrobial efficacy against *E. coli* and *S. aureus* revealed minimal bacterial growth when treated with 5 wt.% ZnO/BCM when compared to the control and BCM blank. However, a higher inhibition of bacterial growth was observed in *S. aureus* than that observed in *E. coli* strains. Observations of the in vivo wound healing effects of 5 wt.% ZnO/BCM on bacterially infected wounds of BALB/c mice reported increased wound size on day 3, which could be linked to bacterial growth and biofilm resistance. However, the wound size significantly decreased to over 90% closure 14 days post-treatment. H&E staining revealed a minimal number of inflammatory cells in 5 wt.% ZnO/BCM-treated groups (Figure 6D). Rayyif et al. designed ZnO-NPs coated polyester-nylon-based nanofibers and noticed that an increase in ZnO-NPs concentration resulted in less agglomeration and uniform covering of the nanofibers by the ZnO-NPs [82]. These nanofibers inhibited the bacterial growth of biofilm-forming pathogens (*S. aureus*, *P. aeruginosa*, *E. faecalis*, and *E. coli*). Planktonic growth also revealed dose-dependent activity, as higher concentrations of ZnO-NPs fibers displayed less pathogen growth than those with lower ZnO-NPs concentrations and the control. These scaffolds further inhibited biofilm formation within the first 24 h, and the effect significantly increased after 48 h. The reported scaffolds have promising wound healing features for infected wounds. However, we recommend that the in vitro cell viability and in vivo wound healing potential of the scaffolds be assessed.

Arshad et al. synthesized thiolated chitosan-alginate (TCS/SA-ZnO) bandages loaded with ZnO-NPs for use in preventing postoperative surgical site infection [59]. The ZnO-NPs had a reported average particle size of 61 ± 9.29 nm with a 0.320 ± 0.01 polydispersity index (PDI) and +20.27 ± 2.92 meV Zeta potential. Loading the bandages with ZnO-NPs significantly increased the porosity, swelling behavior, and tensile strength of the bandages, and the cumulative drug release of ZnO-NPs was sustained for 3 days, which was best fitted to Korsmeyer-Peppas kinetics. A cell viability above 80% for Hela cells was reported after treatment with TCS/SA-ZnO bandages, despite the differences in concentration on the bandages. They evaluated the in vitro antibacterial efficacy of the bandages using the disc diffusion method (DNAse, catalase, and coagulase) and discovered that approximately 9.8 mm of inhibition on Staphylococcus bacteria was exhibited by the bandages compared to 2.1 mm presented by groups treated with the control. The in vivo wound healing potential of the bandages used on the Murine mice model revealed more than 50% wound closure on day 8 post-treatment compared to the marketed bandages, control, and non-thiolated CS/SA-ZnO bandages, which exhibited less than 20% wound closure. On day 16 post-treatment, groups treated with the TCS/SA-ZnO bandages displayed more than 90% wound closure, while other treatment groups still showed less than 50% wound healing.

**Figure 7:**
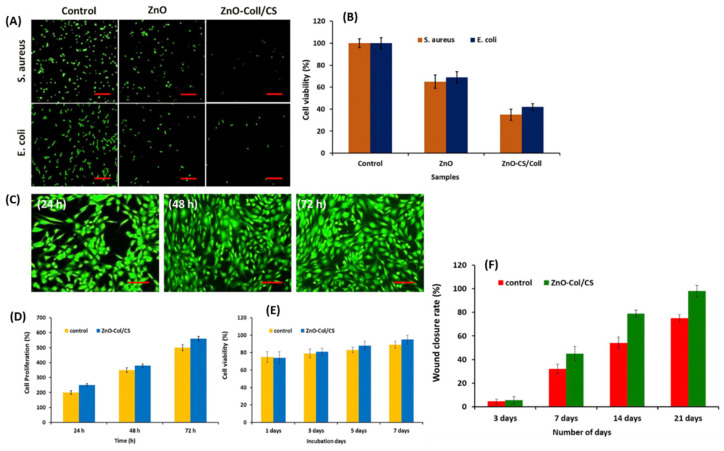
(**A**) Fluorescence microscopic images of *E. coli* and *S. aureus* after treatment with the control, ZnO, and Col/CS-ZnO (scale bar = 50 µm). (**B**) Graph displaying cell viability of the bacterial stains after treatment. (**C**) Fluorescence microscopic images of 8human skin fibroblast cells treated with the nanofibers (scale bar = 100 µm). (**D**,**E**) In vitro cell proliferation and viability of human fibroblast cells after treatment with the nanofibers at different time intervals. (**F**) Graph displaying in vivo wound closure of groups treated with the control compared to the nanofiber treated groups from day 3–21. Adapted with permission from Elsevier [83].

Sun et al. reported an in vivo complete wound healing effect presented by collagenous/chitosan (Col/CS) nanofibers loaded with ZnO-NPs within 21 days post-treatment [83]. The nanofibers induced in vitro cell proliferation and protected human skin fibroblast cells (Figure 7C–E). H&E staining further confirmed that burn wounds treated with Col/CS-ZnO nanofibers exhibited complete re-epithelialization, much more densely-packed keratinocytes, and deposition of collagen types I and III after 21 days. The reported nanofibers also presented higher antibacterial efficacy against *S. aureus* than against *E. coli*; however, the differences were not statistically significant. In a randomized clinical experiment by Loera-Valencia et al. on patients suffering from type 2 diabetes with diabetic foot ulcers, 75% wound closure was achieved after 10 weeks of treatment with calcium alginate-based bandages loaded with ZnO-NPs [84]. These dressings were changed often (every 48 h) to ensure better tissue regeneration and exposure of the nanoparticles within the affected area. The findings reported in this paper suggest that polymeric dressings loaded with ZnO-NPs can promote wound healing while antagonizing bacterial growth and complications associated with type 2 diabetes.

Polymer-based wound dressings loaded with ZnO-NPs, as reported in this section, are promising scaffolds for the management of bacterially infected wounds. However, it must be noted that these dressings are more potent against Gram-positive pathogens than against gram-negative microbes. Their biofilm penetration ability allows them to heal heavily infected and non-healing wounds at an accelerated rate. Nevertheless, frequent dressing changes are required to achieve this, and the concentration of the ZnO-NPs must be taken into consideration for optimum antibacterial activity and cell safety of the dressings. Additionally, the wound healing effect of these dressings is promoted by a synergistic effect between the polymeric material and the ZnO-NPs.

### 4.2. Bioactive Dressings Loaded with Silver Nanoparticles

**Figure 8:**
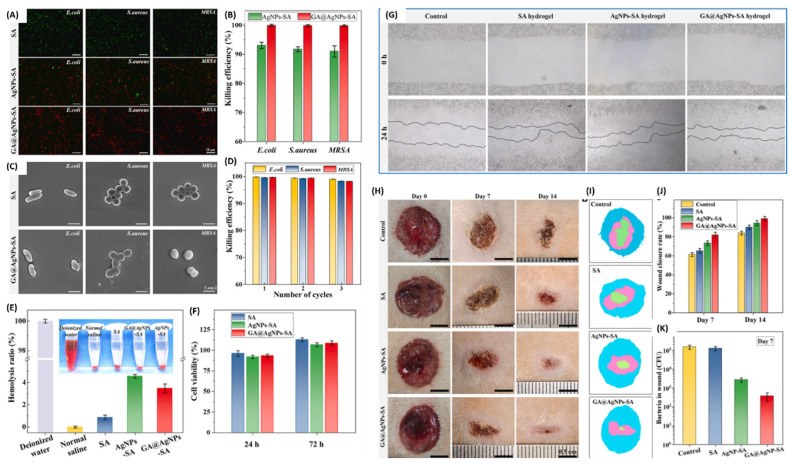
(**A**–**D**) Antibacterial activity of SA-GA@AgNPs, SA-AgNPs, and SA hydrogels: at 20 µm scale bar (**A**) and 1 µm (**C**). (**E**) In vitro haemolysis ratio and (**F**) cell viability of cells after treatment with different dressings. (**G**) In vitro wound scratch assay of L929 cells after treatment with the hydrogels from 0–24 h. (**H**–**K**) In vivo wound healing properties of the hydrogels in different days, showing wound closure and inhibition of bacteria at the wound site. Permission adapted from Elsevier [85].

Hu and co-workers developed sodium alginate (SA) injectable hydrogels that incorporated gallic acid-functionalized AgNPs (GA@AgNPs) to foster rapid wound closure of microbially infected wounds [85]. The hydrogels showed high swelling ratios of ±2886% and high elongation at breaking point, with an initial burst release of AgNPs (±60%) in 2 days and a sustained release of up to 7 days. In vitro antimicrobial activity revealed a minimal bacterial survival rate (±1%) for MRSA, *E. coli*, and *S. aureus* when incubated with SA-GA@AgNPs hydrogels as opposed to the survival rate when exposed to SA-AgNPs hydrogels (8%) (Figure 8B). The hydrogels also significantly reduced the thickness of biofilms and decreased the mass of bacteria forming pathogens. The high cell viability of L929 after co-culture with the hydrogels allowed the researchers to use them for further studies. In vitro wound scratch assay revealed ±85% wound closure within 24 h across all the treatment groups (SA-GA@AgNPs, SA-AgNPs, and SA hydrogels) except for the control group, in which the wound closure was only ±60% (Figure 8G). In vivo wound healing assays of SA-GA@AgNPs were investigated on a Sprague-Dawley MRSA-infected full-thickness skin defected rat model, and a 99.1% wound closure was achieved by day 14 with less than 10^3^ colony-forming units (CFU/mL) on day 7 (Figure 8H–K). H&E and Masson’s trichrome staining revealed fewer neutrophils and inflammatory cells in the SA-GA@AgNPs treatment groups.

Zhou et al. developed CS sponges (CS-it/AgNPs) incorporated with iturin-AgNPs and evaluated their in vitro and in vivo wound healing abilities [86]. The reported iturin-AgNPs revealed a spherical morphology 20 ± 10 nm in diameter. The in vitro antibacterial activity of different formulations of CS-it/AgNPs was evaluated against *E. coli* and *S. aureus*, and it was observed to increase with an increase in the concentration of AgNPs. However, the highest antibacterial efficiency of the scaffolds was observed against *E. coli,* with ±22 cm of inhibition. *S. aureus* was inhibited to ±9 cm under the same conditions. The in vivo wound healing properties of CS-it/AgNPs sponges were evaluated on infected Kunming male mice and compared to that of control and AgNPs-gauze. The mice treated with CS-it/AgNPs sponges displayed complete wound healing by day 16, with hair follicles growing around the wound site. Similar results were observed for the AgNPs-gauze treated groups, while ±78% wound closure was recorded for the control groups by day 16.

Polyethylene glycol diacrylate/hyaluronic acid double network hydrogels loaded with silver-doped silica nanoparticles were developed by Huang et al. [87]. These hydrogels displayed a consistent in vitro antibacterial efficacy against *S. aureus* and *E. coli*, which could be linked to their sustained release of Ag from 3 h to day 18. The reported hydrogels further displayed no signs of toxicity against Human Umbilical Vein Endothelial Cells (HUVECs) in 5 days of incubation, and their wound healing potential was further evaluated in vivo against bacterially inoculated full-thickness wounds (7 mm in diameter) of female Sprague-Dawley mice. The hydrogels displayed about 75% wound closure within 6 days, and a complete wound closure was observed in 12 days, with hair follicles fully grown at the wound site. Treatment groups further displayed reduced expression of inflammatory cells (IL-1β and IL-6) compared to the control treatment groups. Ma et al. incorporated AgNPs in polydopamine/polyethyleneimine-coated bacterial cellulose nanofibers and reported their in vitro microbial inhibition against *S. aureus* and *E. coli* [88]. A 100% wound closure of bacterially inoculated wounds (10 mm) in Kunming mice was observed 12 days post-wound infection when the mice were treated with the nanofibers. These nanofibers also reduced the inflammatory response by decreasing the expression of TNF-α and IL-1β at the wound site, as observed under H&E staining.

**Figure 9:**
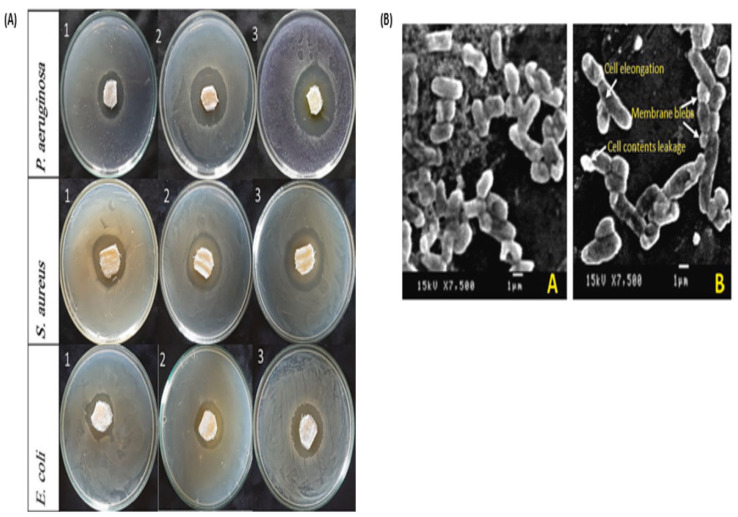
(**A**) Inhibition zones exhibited by the dressings loaded with different concentrations of Ag-NPs; (1) 8.3 μg/mL, (2) 16.6 μg/mL, and (3) 33.25 μg/mL against *E. coli* ESC3, *S. aureus* STA7, and *Paeruginosa* Pau. (**B**) SEM images displaying morphology of *P. aeruginosa* Pau before and after incubation with the dressing; intracellular content leakage, membrane damage, and membrane blebs were observed after treatment. Permissions adapted from Elsevier [89].

Hamdy et al. reported high antimicrobial efficacy exhibited by polyhydroxybutyrate/gelatin-based dressings loaded with AgNPs against *S. aureus* STA7, *P. aeruginosa* Pau, and *E. coli* ESC3 [89]. The antibacterial activity of these scaffolds increased with an increase in the concentration of AgNPs (Figure 9A). Jiang and co-workers designed a sodium alginate/chitosan-based sponge loaded with sericin, AgNPs, and different concentrations of curcumin for the treatment of infected wounds [90]. The reported sponges displayed swelling properties above 1000%, showing their potential for diffusion of nutrients, absorption of wound exudate, and drug release kinetics. The sponges further displayed in vitro biocompatibility against HUVECs, HaCaT cells, and blood cells with a haemolysis rate of less than 5%. The in vitro antibacterial efficacy against the biofilm-forming strains (*P. aeruginosa* and *S. aureus*) displayed by the sponges motivated their further application in vivo to infected male Sprague-Dawley mice with wounds 10 mm in diameter. A 92% and 97% wound closure were recorded on full-thickness defects inoculated with *P. aeruginosa* and *S. aureus*, respectively, on day 12 post-treatment. The H&E staining of groups treated with SA/CS-Ser/Cur/AgNPs displayed improved epithelial regeneration, higher expression of CD31, and lower levels of inflammatory cells (TNF-α).

Silver-loaded electrospun nanofibers displayed high in vitro cell safety in L929 mouse fibroblast cells after incubation for 24 h [91]. The loading of AgNPs synergistically promoted in vitro microbial inhibition of *P. aeruginosa* and *S. aureus*, which led to their in vivo wound healing evaluation on a 1 cm round wound incised on albino mice. The reported electrospun nanofibers revealed no signs of tissue adhesion or skin re-damage when removed from the wound site, and the mice exhibited about 96% wound closure on day 13 post-injury. Ohta et al. formulated carboxymethyl cellulose-based (CMC-AgNPs) nonwoven sheets loaded with AgNPs for the treatment of infected wounds [92]. These dressings displayed high antibacterial activity against *P. aeruginosa* and *S. aureus* and a moderate inhibition of *E. coli*. The antibacterial efficacy of these scaffolds was linked to the release kinetics of AgNPs. In vivo wound healing of pressure-ulcer bacteria-inoculated mice wounds revealed faster wound closure 25 days post-infection when treated with CMC-Na/AgNPs than when treated with commercially available Aquacel Ag^®^.

**Figure 10:**
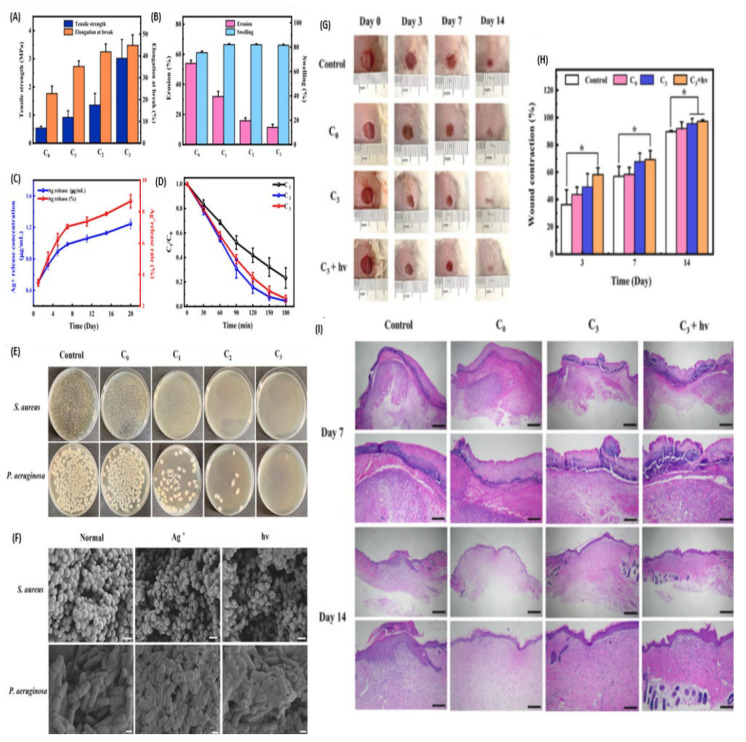
(**A**–**D**) Mechanical properties of the prepared composite films. (**E**) In vitro antibacterial activity of the formulations based on the concentration of C3N4-PDA-Ag (0, 0.5, 1.5, 2.5, respectively, for C_0_, C_1_, C_2_, and C_3_). (**F**) SEM images of *S. aureus* and *P. aeruginosa* after treatment. (**G**) Wound healing images of mice after treatment from day 0–7 post-injuries. (**H**) Quantitative wound closure exhibited by different dressings; * *p* < 0.05. (**I**) H&E staining images of control, C_0_, C_3_, and C_3_ + hv groups on days 7, and 14. Adapted with permission from Elsevier [93].

Liu et al. evaluated the wound healing potential of chitosan-based (CS-AgNPs/C_3_N_4_-PDA) dressings loaded with AgNPs and carbon nitride-polydopamine as antimicrobial agents [93]. In vitro antibacterial assays displayed a low bacterial survival rate when the dressings had been treated with CS-AgNPs/C_3_N_4_-PDA, and this was influenced by an increase in the concentration of AgNPs and C_3_N_4_. SEM images of *S. aureus* and *P. aeruginosa* after treatment with CS-AgNPs/C_3_N_4_-PDA revealed raptured cell membranes with pores, signifying the antibacterial mechanism of action for AgNPs and that of C_3_N_4_ is associated with the generation of reactive active species under visible light (Figure 10F) [93]. These scaffolds displayed haemocompatibility and biocompatibility against blood cells and L929 cells in vitro, and their in vivo wound healing potential was evaluated on *S. aureus*-infected wounds (8 mm in diameter) of ICR mice. The treatment groups treated with visible light displayed 97.25% wound closure by day 14, followed by a group treated without the presence of visible light at 95.53%, while the blank control displayed 89.67% (Figure 10H). These findings confirm the antibacterial mechanism of action for C_3_N_4_ under visible light and a synergistic effect between the bioactive materials and the polymer matrix. H&E staining confirmed that treatment groups under visible light had a higher degree of re-epithelialization on day 14, with hair follicles and glands visible, and fewer inflammatory cells observed from day 7 onwards.

Choudhary et al. developed chitosan-based hydrogel films (CS-PL/G/AgNPs) loaded with ε-Poly-L-lysine and graphene silver nanoparticles for microbially infected wounds [94]. The hydrogel films presented 99.99% killing efficiency for *E. coli* and *S. aureus* while exhibiting cell viability for whole blood cells and L929 fibroblast cells. They were then evaluated for wound healing both in vitro and in vivo against L929 cells and the Wistar rat model. A 1 mm wound scratch treated with CS-PL/G/AgNPs healed within 18 h, and a 2 × 2 cm wound area on a Wistar rat displayed 94.12 ± 2.65% wound closure on day 14 post-infection. The in vivo wound healing potential of these hydrogel films was further compared to those of commercially available Tegaderm and Fibroheal@Ag, which displayed 84.59 ± 2.22% and 72.06 ± 2.01% wound reduction after 14 days, respectively. H&E staining also confirmed the complete wound healing, with thick epidermis tissue formed [94]. Kong et al. synthesized Riclin-capped AgNPs that are biocompatible against NIH3T3 cells and noted that an increase in the concentration of AgNPs decreased the cell viability of NIH3T3 cells [63]. These composites exhibited microbial inhibition of both *E. coli* and *S. aureus* in vitro, and wound healing studies on male C57 BL/6 mice with *S. aureus*-inoculated wounds (1 cm) displayed 100% wound closure after 12 days. The H&E stain made after 8 days displayed intact epidermis, the accumulation of keratinocytes, lower levels of pro-inflammatory factors (IL-1β, IL-6, TNF-α), and complete re-epithelialization.

Chu et al. developed sericin-based hydrogels incorporated with lupeol-loaded chitosan-Ag^+^ nanoparticles and investigated their in vitro and in vivo wound healing activity [95]. The hydrogels displayed haemocompatibility with a less than 5% haemolytic ratio. Nevertheless, they exhibited bacterial cell toxicity against both Gram-positive and gram-negative strains of bacteria. The hydrogels exhibited controlled drug release, biodegradation, tensile strength, and swelling ratios (>1200%). Two cm wound incisions on Sprague-Dawley rats were infected with *E. coli* and *S. aureus* and later treated with the gel formulation. The hydrogel treated groups displayed 95.11% wound closure after 17 days and a complete wound healing in 21 days. Moreover, the hydrogel treated groups revealed uniform coloration, neatly arranged collagen fibers, and decreased inflammatory cells.

### 4.3. Bioactive Dressings Loaded with Gold Nanoparticles

**Figure 11:**
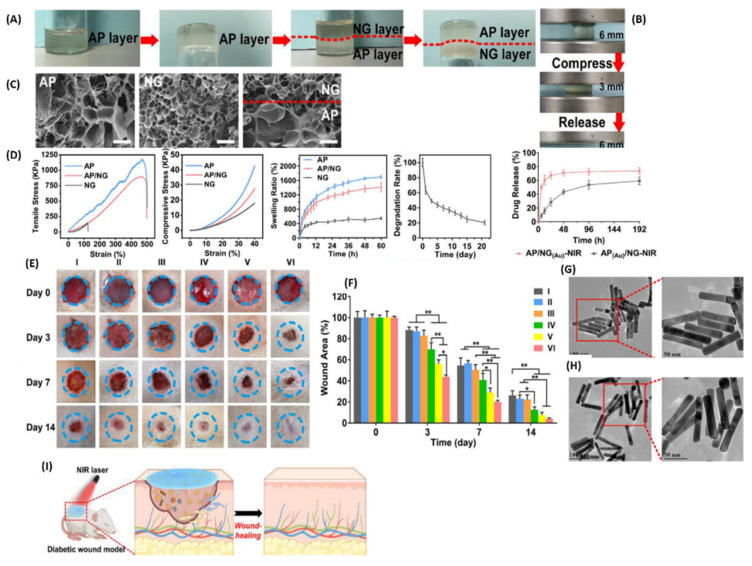
(**A**) Photographs of the double-layered hydrogel. The red dashed line signifies the top layer from the bottom layer. (**B**) Compression and recovery of the hydrogels. (**C**) SEM images of the single layer and double-layer hydrogel. (**D**) Mechanical properties of hydrogels. (**E**) Display photographs of the wound area after treatment at different time intervals. (**F**) Quantitative wound reduction at different time intervals. (**G**,**H**) TEM images of Au-nanorods and peptide-functionalized Au-nanorods. (**I**) Graphic presentation of the hydrogel and its diabetic wound healing process. *n* = 3, * *p* < 0.05, ** *p* < 0.01. (For Permissions adapted from Elsevier [96]).

A study utilizing near-infrared light-responsive double-layer hydrogels loaded with peptide-functionalized Au-nanorods showcased accelerated wound healing in type 2 diabetic Sprague-Dawley rats within 14 days (Figure 11) [96]. The top layer exhibited higher swelling ratios (1600%) compared to the lower layer (500%), while the average ratios for the double-layer hydrogels was 1400%. The lower layer displayed an initial burst release of peptide-functionalized Au-nanorods (AuP) in the first 6 h, which was later sustained until 48 h with a cumulative release of 73.7%. Conversely, the top layer revealed a controlled release for up to 8 days, with a cumulative release of 59.1%. The rapid initial drug release presented by the lower layer that is in contact with the wound site enables it to exert a rapid antibacterial effect in the early inflammatory stages [96]. The hydrogels exhibited the highest killing efficacy against *E. coli* (99%) compared to the *S. aureus* (98%), but there was no significant difference. Additionally, the hydrogels exhibited viability of the HUVECs and promoted cell proliferation, migration, and angiogenesis. Interestingly, the bilayer hydrogels upregulated focal adhesion kinase (FAK), integrin b1 (ITGB1), integrin-linked kinase (ILK), fibroblast growth factor (bFGF), CD31, kinase domain receptor (KDR), and neuropilin-1 (NRP1) [96].

Azlan et al. developed pluronic-F127-based gels loaded with bioactive materials and reported that they induced >85% wound closure of 10 mm wounds on diabetic Wistar rats 14 days after treatment [97]. The gels induced wound healing by promoting rapid increases in blood vessel density, vascular endothelial growth factors (VEGF), VEGF-A levels, CD-31 expression, higher prostaglandin E2 production, and a reduced number of inflammatory cells. The gels further demonstrated high antibacterial efficacy against a range of both gram-negative and Gram-positive strains of bacteria, a key feature in the management of diabetic and infected wounds. The wound healing features exhibited by the gels were significantly better than those exhibited by Tegaderm, Intrasite gel, and Hypafix Dressings. The gels exhibited low cohesiveness and hardness and good spreadability, viscosity, and adhesive properties, which made them suitable for use in wound healing without causing pain during removal.

Raghuwanshi et al. synthesized gold nanoparticles from *Woodfordia fruticose* leaves and fabricated them in carbopol^®^934-based gels for the management of microbial infected wounds [98]. The viscosity and spreadability of the gels decreased with an increase in the concentration of Au-NPs loaded. The topical gels displayed antimicrobial activity against *Cryptococcus neoformans* and *Candida albicans* fungal strains. Moreover, they exhibited biofilm inhibition and biofilm eradication. Albino Wistar rats were used to investigate the in vivo wound healing effects of the formulations. Other treatment groups were untreated, treated with carbopol^®^934 ointment, and treated with Povidone iodine. After 12 days, accelerated wound healing was observed in groups treated with carbopol^®^934/Au-1% (96.01 ± 0.21 mm^2^) and Povidone iodine (93.80 ± 0.15 mm^2^), compared to carbopol^®^934/Au-2% (81.79 ± 0.22 mm^2^), carbopol^®^934 ointment (69.18 ± 0.27 mm^2^), and untreated (67.20 ± 0.26 mm^2^).

Photothermal hydrogels containing gold nanoparticles embedded in halloysite nanotubes exhibited high bacterial killing efficacy against *S. aureus* (98%) and *E. coli* (99%). The wound healing characteristics of the hydrogels were linked to near-infrared light irradiation [99]. The hydrogels revealed good elasticity with elongation at break point and tensile strength of 151.7% and 137.4 KPa, respectively. The hydrogels further presented more than 50% swelling ratios after 45 h. The haemolysis rate of RBCs was recorded at 3% when incubated with the hydrogels, showing their safety for application in wound healing. The in vivo wound healing of a mouse model with a full-thickness defect wound showed more than 95% wound closure after 14 days of treatment with the hydrogels. The hydrogel treated groups revealed complete wound healing in 21 days, with light scarring observed at the healed area. H&E staining showed decreased inflammatory cells, formation of epidermis, and granulation tissue for the hydrogel treated groups on day 14 post injury.

### 4.4. Bioactive Dressings Loaded with Magnesium Oxide Nanoparticles

Bhattacharya and co-workers developed PVA/PEG hydrogels loaded with human epidermal growth factors (HEGF) and 1% or 5% concentration of MgO-NPs [100]. The porosity and water-vapor transmission rate of the hydrogels decreased with an increase in concentration of MgO-NPs. Hydrogels loaded with 5% MgO-NPs (H_5) displayed the highest water content and swelling ratios. The incorporation of MgO-NPs led to an increased elasticity of the hydrogels, with improved tensile strength and elongation at break point. Antibacterial assays displayed the lowest colony counts (CFU/mL) of *P. aeruginosa* and *B. subtilis* when treated with H_5. The in vitro viability of a human dermal fibroblast cell line was highest when treated with hydrogel formulations containing both MgO-NPs and HEGF. A surgical wound size of about 1 cm on Wister rats was used to investigate the wound healing properties of Tegaderm, H_5, PVA/PEG (H_0), PVA/PEG-HEGF (H_0_E), PVA/PEG-5% MgO/HEGF (H_5_E), and no treatment. The Tegaderm and H_5_E treatment groups displayed the most efficient wound reduction after two weeks, with almost complete wound healing, compared to other treatment groups, which remained infected with no visible healing.

**Figure 12:**
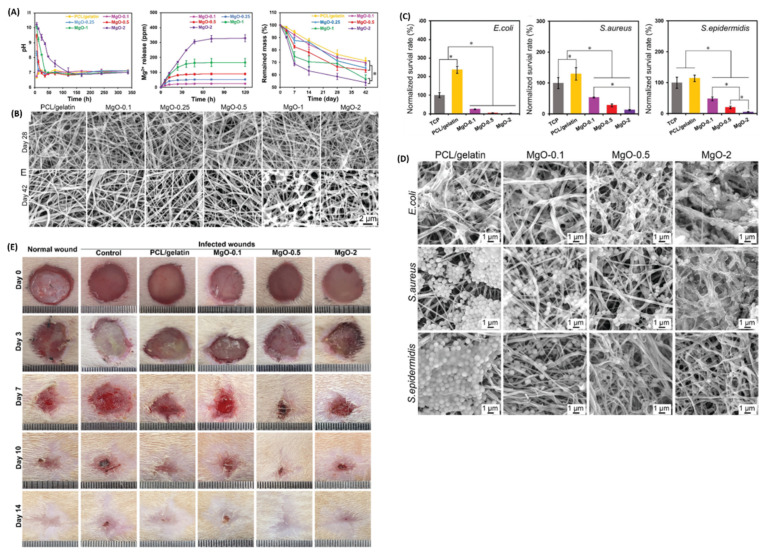
(**A**) Mechanical properties of the nanofibers. (**B**) SEM images of the nanofibers loaded with different concentrations of MgO-NPs. (**C**) In vitro antibacterial efficacy of the nanofibers. (**D**) SEM images of *E. coli*, *S. aureus*, and *S. epidermidis* after treatment with different dressings, showing mass death and debris. (**E**) Wound healing process after treatment with different wound dressings in different time frames (*n* = 4, * indicates *p* < 0.05). Adapted with permissions from The Royal Society of Chemistry [101].

Polycaprolactone/gelatine-nanofiber membranes exhibited antibacterial efficacy (90% *S. aureus*, 94% S. epidermidis, and 98% *E. coli*) that is directly proportional to an increase in the concentration of MgO-NPs incorporated [101]. However, higher concentrations of MgO-NPs suppressed the proliferation of HUVECs and NIH 3T3 fibroblasts. Nevertheless, the reported membranes displayed cell safety, good tensile strength, and flexibility, making them suitable for in vivo wound application. A 7 mm wound diameter of Sprague-Dawley rats was infected with 20 µL *S. aureus* suspension and later treated with a 3M Adhesive Bandage and nanofiber membranes incorporated with 0.1%, 0,5%, and 2% MgO-NPs, denoted as PCL/Gel-0.1, PCL/Gel-0.5, and PCL/Gel-2, respectively. The wound exudate was collected on all treatment groups after 3 days of treatment and using the Mueller-Hinton broth. It was noted that PCL/Gel-0.1 did not inhibit the bacterial growth of *S. aureus.* The other treatment groups showed about 50% inhibition. However, on day 14, all treatment groups exhibited more than 75% wound closure, with PCL/Gel-0.5 treated groups showing about 98% wound healing followed by the normal wound groups (non-infected) with about 92% (Figure 12E). The normal wound groups presented faster wound closure than the other treatment groups (with the exception of the PCL/Gel-0.5 group), which made it difficult to distinguish the biological activity of the prepared nanofiber membrane formulations [101]. Nevertheless, the histological assessment showed hair follicles, blood vessels, mature epidermis, and organized fibroblasts for PCL/Gel-0.5 treated wounds. These findings suggest that the immune response of the Sprague-Dawley rats played a significant role in inhibiting bacterial invasion and thus accelerating normal wound healing cascades.

In another study by Liu et al., they used PCL/Gel-0.5 for treating (10 mm diameter) wounds in diabetic rats. They noted that the membranes promoted healthy blood vessel formation one week post-surgery [102]. These membranes further displayed a notably accelerated healing process of diabetic wounds by reducing inflammation, encouraging the growth of new blood vessels, and enhancing the formation of granulation tissue. The nanofiber membranes induced rapid wound healing by upregulating the M1 to M2 switch of macrophages, alleviating the inflammatory response, and enhancing the formation of granulation tissue. Eivazzadeh-Keihan and co-workers developed carboxymethyl cellulose-based hydrogels incorporated with silk fibroin and Mg(OH)_2_-NPs for the management of infected wounds [103]. These hydrogels displayed a tensile strength of 299.35 to 250.78 MPa, Young’s modulus (11.34 to 10.14 Mpa), swelling ratio (7%), hydrophilicity, elongation at break point (12.52 to 12.84%), and water uptake capacity of 92.5%. These features improved as the accumulation of Mg(OH)_2_-NPs increased. The porosity of the hydrogels decreased with an increase in the amount of silk fibroin loaded [103]. The hydrogels further presented in vitro haemocompatibility of RBC and biocompatibility of Hu02 cell lines, with 8.3% haemolytic effect and 84.5% cell viability, respectively. Their high antibacterial activity was observed against *P. aeruginosa,* and they significantly constrained biofilm formation, exhibiting the lowest OD values (0.13). The in vivo wound healing of mice displayed rapid wound closure when treated with the hydrogels (82.29%) compared to the control (no dressing) groups, which had a wound closure of 75.63% after 12 days. 

**Figure 13:**
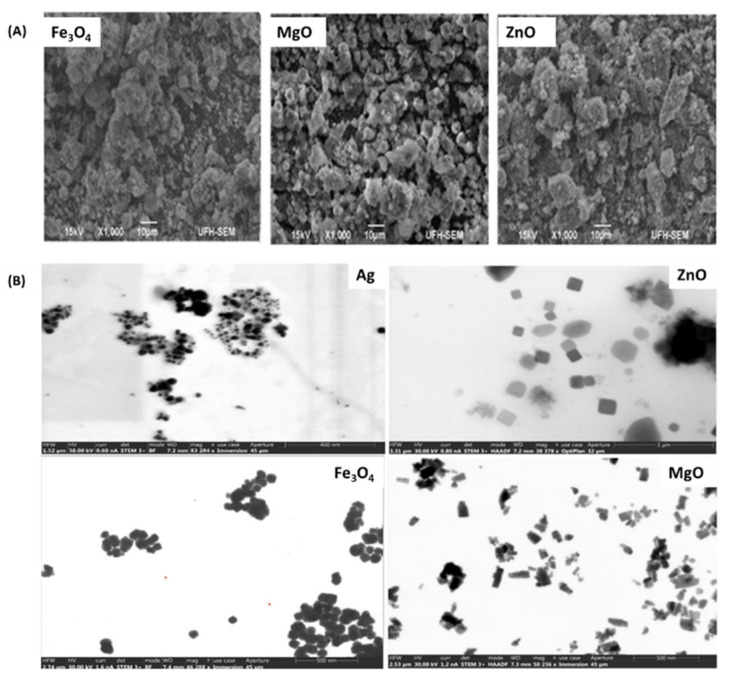
(**A**) SEM images of the nanoparticles. (**B**) TEM photographs different nanoparticles. Permission adapted from Springer Nature [104]. Copyright link https://creativecommons.org/licenses/by/4.0/ (accessed on 30 October 2023).

Sodium alginate-based topical gels loaded with tranexamic acid and a variety of metal-based nanoparticles (MgO, ZnO, Ag, and Fe_3_O_4_) displayed high in vitro wound healing effects [104]. The SEM and TEM images of the nanoparticles are displayed in Figure 13 below. These topical gels exhibited good spreadability and viscosity, suggesting a pseudoplastic behavior and showing their potential to remain at the wound site after application without flowing off. The reported gel formulations exhibited a broad antibacterial spectrum against both Gram-positive and gram-negative strains of bacteria. The in vitro cytotoxicity studies revealed a concentration-dependent cell viability of HaCaT cells when incubated with the gel formulations after 24 h. Nevertheless, the reported gel formulations exhibited cell safety at a concentration of 100 µM and lower. The in vitro whole blood clot assay revealed that the topical gel formulation can potentially promote rapid blood coagulation compared to the control, with a *p*-value of (0.0014–0.0121) Additionally, an in vitro wound scratch assay displayed rapid wound closure for a topical gel containing MgO and AgNPs, with ±88% and ±81% after 72 h of incubation, respectively. The control group showed a wound closure of 42%. The reported findings suggest that topical gels containing MgO and AgNPs can be applied for the management of infected wounds. However, in vivo biological studies still need to be reported to confirm the potential clinical application of the gels.

Sukumaran and co-workers synthesized chitosan/gelatine composite films loaded with different concentrations of MgO-NPs (1, 2, and 3%) and evaluated their in vitro wound healing effects [105]. The biocomposite films displayed swelling degrees between 1075 and 854%, with porosities and average pore diameters in the ranges of 57–78% and 73–103 µm, respectively. An increase in the concentration of MgO-NPs decreased the swelling and porosity of the films, which reduced the tensile strength from 1.74 to 1.53 MPa. The films further displayed biodegradability and biocompatibility with C3H10T1/2 cells. Moreover, high antibacterial activity of the films against *E. coli* and *S. aureus* with zones of inhibition recorded at 13 and 12 mm for MgO-1%, 16 and 14 mm for MgO-2%, and 21 and 20 mm for MgO-3%. The chitosan/gelatine films with MgO-1% were used to investigate the in vitro wound scratch healing of C3H10T1/2 cells. Chitosan/gelatine/MgO-1% treated groups displayed complete wound closure in 12 h.

### 4.5. Bioactive Dressings Loaded with Bimetallic Nanoparticles

Saravanakumar et al. reported the in vitro wound healing potential of xanthan gum nanocomposites incorporated with a combination of AgNPs, MgO-NPs, and aloe vera extracts [106]. The nanocomposites exhibited antibacterial inhibition against *E. coli* and *B. cereus*, showing 14.5 ± 0.85 mm and 15 ± 0,12 mm zones of inhibition and MIC values of 2.5 µg/mL and 0.62 µg/mL, respectively. Moreover, higher biofilm eradication was observed after treatment with the nanocomposites. SEM images showed disrupted surface morphology and a change in the shape of the remaining biofilm. The antibacterial activity of these nanocomposites was linked to the synergistic effect of the bimetallic nanoparticles (Ag/MgO), xanthan gum, and aloe vera. The reported samples further displayed non-toxicity against RBCs, NIH3T3, and HEK293 cells. Additionally, an in vitro wound scratch assay using a 200 µL micropipette tip on NIH3T3 cells revealed 91% wound closure in 48 h when treated with the nanocomposites, compared to other treatment groups, which showed less than 75% wound closure. The reported scaffolds displayed promising wound healing effects. Nevertheless, in vivo biomedical studies of the materials are still needed to check their eligibility and potential application in clinical studies. Gouda et al. developed polymeric films incorporating MgO and CuO-NPs as potential wound dressing materials [107]. The materials showed good contact angles and their swelling ratios decreased with an increase in the bimetallic nanoparticles added. The films further showed good biodegradability after 35 days and exhibited high cell safety (80%) after incubation with human osteosarcoma MG-63 cells. Additionally, these scaffolds exhibited a high cell proliferation of 122%. The reported scaffolds are potential wound dressing materials, but further studies are required both in vitro and in vivo to confirm their wound healing ability. 

Arumugam et al. developed silk fibroin/gelatine nanofibers loaded with Ag and Au-NPs to promote rapid wound healing by reducing inflammatory response [108]. The nanofibers displayed cell viability of L929 cells at different dose concentrations. However, they also increased apoptosis by 11–29%, dependent on dose concentration. The nanofibers induced wound healing in Sprague-Dawley rats in 21 days. The treated groups displayed hair follicles and newly formed blood vessels on the healed area. The accelerated wound healing promoted by the nanofibers is linked to their biocompatibility, sustained drug release, generation of ROS, and antimicrobial activity.

Bagheri and colleagues synthesized chitosan/polyethylene oxide (CS/PEO) nanofibers incorporated with ZnO and silver nanoparticles for the antibacterial management of infected wounds [109]. The MTT assay of fibroblast cell viability displayed a dose-dependent survival rate that decreased as the concentration of the nanoparticles increased. It should be noted that nanofibers containing AgNPs presented higher cell safety compared to nanofibers containing ZnO-NPs. This was also observed in the antioxidant activity of the nanofibers. Nanofibers loaded with both nanoparticles also showed high cell safety and an antioxidant activity better than that of CS/PEO-ZnO nanofibers. The in vitro tests of antibacterial efficacy against Gram-positive *S. aureus* revealed the highest zone of inhibition when treated with CS/PEO-ZnO/AgNPs (~35 mm), followed by CS/PEO-ZnO (~21 mm) and CS/PEO-AgNPs (~13 mm). By contrast, when tested against the gram-negative strains (*P. aeruginosa* and *E. coli*), these scaffolds revealed minimal antibacterial activity compared to the control, with CS/PEO-ZnO/AgNPs exhibiting ~11 mm and 14 mm, CS/PEO-AgNPS ~11 and 10 mm, and CS/PEO/ZnO ~9 and 10 mm, respectively. An in vitro wound scratch assay displayed no statistical difference in wound closure across all treatment groups, with CS/PEO-ZnO, CS/PEO-AgNPs, and CS/PEO-ZnO/AgNPs all displaying ± 70% wound healing after 24 h.

Lu et al. developed chitosan-based dressings incorporated with hybrid Ag/ZnO-NPs for the treatment of bacterially inoculated wounds [110]. These scaffolds exhibited a high antimicrobial effect against *E. coli*, *P. aeruginosa*, and *S. aureus*, which increased with an increase in the concentration of ZnO-NPs. Bacterial inhibition was also observed against drug-resistant strains of *E. coli* and *S. aureus* at higher concentrations. However, an increase in the concentration of ZnO-NPs had no significant impact on drug-resistant *P. aeruginosa*, which displayed no zone of inhibition. The increase in concentration of ZnO-NPs led to a decrease in the cell viability of L02 cells. The wound (7 mm) healing test using BALB/c mice revealed more than 90% wound closure on day 7 compared to treatment groups with only ZnO-NPs. These findings suggest that the polymeric network and hybridization of ZnO-NPs and AgNPs synergistically accelerate wound healing. H&E staining revealed packed keratinocytes and an organized granulation tissue after 10 days.

The ZnO/AgNPs hybrid-loaded xerogels displayed 99.9% and 99.85% bacterial killing effects against *E. coli* and *S. aureus*, respectively [111]. The high antibacterial inhibition exhibited by these dressings was linked to high ROS generation inside the bacteria. The NIH3T3 cells displayed high cell viability after incubation with the xerogels, and in vivo wound healing was evaluated on male Wistar rats with a 12 mm wound incision. Complete wound healing was observed after 10 days of treatment with the xerogels, and the H&E and Giemsa staining presented fewer inflammatory cells and cellular vacuolation with normal tissue were visually observed. Kantipudi et al. developed ZnO/AgNPs-loaded composite gels and investigated their wound healing potential on infected Wistar Albino rats [112]. The reported gels displayed a 95 ± 3.53% wound reduction on day 10 post injury, which was significantly higher than that of treatment groups treated with only ZnO-NPs or AgNPs.

The encapsulation of hybrid metal nanoparticles significantly enhanced wound reduction and displayed an improved bacterial killing effect. However, the issue of the concentration of ZnO-NPs should always be taken into consideration for relative cell safety. Nevertheless, the two metal-based nanoparticles displayed a synergistic wound-healing effect (Table 1). Further, when only one nanoparticle is encapsulated on a bioactive dressing, a trend of higher efficacy against either gram-negative or Gram-positive bacteria is observed. This trend is not as obvious in hybrid-loaded dressings. 

## 5. Conclusions and Future Perspectives

In conclusion, the advancements in wound dressing technologies, particularly those integrating metal-based nanoparticles into bioactive dressings, represent a significant stride toward effective wound management and tissue regeneration, especially in microbially infected and chronic wounds. Polymer-based dressings, with their remarkable properties (high swelling, porosity, and controlled drug release), combined with the inclusion of metal-based nanoparticles, demonstrate a synergistic effect that enhances their therapeutic potential. The controlled release of nanoparticles within these dressings plays a critical role in their antibacterial activity, causing cellular damage, inducing ROS, disrupting microbial protein synthesis, and impeding DNA and RNA replication. The ability of metal-based nanoparticles to penetrate resistant biofilms, inhibit bacterial growth, and interfere with vital bacterial mechanisms highlights their efficacy in wound healing. The reported wound dressing scaffolds exhibit promising attributes, showcasing potential for future clinical trials. With their multifaceted approach addressing microbial infection and supporting tissue regeneration, these advancements pave the way for innovative and effective strategies in wound care, offering hope for improved outcomes in the treatment of microbially infected and chronic wounds.

## Figures and Tables

**Table 1:** Summary of bioactive dressings loaded with metal-based NPs.

Wound Area	Healing Time	Bioactive Dressing	NPs	Role in Wound Healing	Ref.
-	16 days	Bandages	ZnO-NPs	Antibacterial efficacy, cytocompatibility, controlled drug release, promote wound healing.	[59]
8 mm	14 days	Biocomposites	ZnO-NPs	Concentration-dependent antibacterial activity, antioxidant, antibiofilm effect, and wound healing.	[79]
2 × 2.5 cm	14 days	Nanofibers	ZnO-NPs	Antibacterial (*E. coli* and *S. aureus*), cytocompatibility, synergistic wound healing effect, collagen deposition and re-epithelialization.	[80]
6 mm	14 days	Membranes	ZnO-NPs	Antibacterial, biocompatible, wound healing effects, and reduce inflammatory cells.	[81]
7 mm	21 days	Nanofibers	ZnO-NPs	Antibacterial efficacy, and wound healing, promoted complete re-epithelialization, much denser packed keratinocytes and deposition of collagen type I and III.	[83]
**-**	10 weeks	Bandages	ZnO-NPs	Promote wound healing of type 2 diabetic patients and antagonizing bacterial growth.	[84]
**-**	48 h		AgNPs	Promoted in vitro upregulation of growth factors (VEGF and PDGF), crucial in wound healing processes, biocompatible.	[60]
1 cm	12 days	Hydrogels	AgNPs	Exhibited microbial inhibition, cell viability, and wound healing with intact epidermis, accumulation of keratinocytes, lower levels of pro-inflammatory factors, IL-1β, IL-6, TNF-α, and a complete re-epithelialization.	[63]
1.2 cm	14 days	Hydrogels	AgNPs	Antimicrobial activity, biofilm degradation, high swelling ability, sustained drug release, improve tissue regeneration, and lowers levels of neutrophils and inflammatory cells such as TNF-α and IL-6.	[85]
7 mm	16 days	Sponges	AgNPs	Microbial inhibition, cytocompatibility, promoted rapid tissue regeneration with visible hair follicles.	[86]
7 mm	12 days	Hydrogels	AgNPs	Antimicrobial activity, sustained drug release, biocompatibility, promoted wound healing, and displayed reduced expression of inflammatory cells (IL-1β and IL-6)	[87]
10 mm	12 days	Nanofibers	AgNPs	Bacterial growth inhibition, cytocompatibility, displayed progressive wound healing, and decreased expression of TNF-α and IL-1β cells at the wound site.	[88]
10 mm	12 days	Sponges	AgNPs	Antibacterial efficacy, high swelling properties, biocompatibility, haemocompatibility, rapid wound closure, and improved epithelial regeneration, higher expression of CD31, and lower levels of inflammatory cells (TNF-α).	[90]
1 cm	13 days	Nanofibers	AgNPs	Synergistic antibacterial effect, biocompatible, and improves tissue regeneration.	[91]
12 mm	25 days	Nonwoven sheets	AgNPs	Inhibits bacterial growth, sustained release kinetics, cytocompatibility, and promotes wound healing.	[92]
8 mm	14 days	Composite films	AgNPs	Antioxidant activity, haemocompatibility, biocompatibility, induce wound reduction, and promotes higher degree of re-epithelialization with hair follicles and glands visible, and decreased inflammatory cells.	[93]
2 × 2 cm	14 days	Hydrogels	AgNPs	Exhibit high bacterial killing efficiency, cell viability, and complete wound healing with thick epidermis tissue formed.	[94]
6 mm	7 days	hydrogels	Au-NPs	Promote rapid wound closure, antibacterial efficacy, haemocompatible, and biocompatible.	[68]
10 mm	14 days	hydrogels	Au-NPs	Antimicrobial, upregulated focal adhesion kinase (FAK), fibroblast growth factor (bFGF), CD31, kinase domain receptor (KDR), and neuropilin-1 (NRP1.	[96]
10 mm	14 days	Gels	Au-NPs	Promote rapid healing of diabetic rats, promote rapid blood vessel density, vascular endothelial growth factors (VEGF), VEGF-A levels, CD-31 expression, and reduced number of inflammatory cells, antibacterial efficacy.	[97]
-	12 days	Gels	Au-NPs	Antimicrobial activity, biofilm inhibition and biofilm eradication, wound healing of infected wounds.	[98]
	14 days	Hydrogels	Au-NPs	High bacterial killing efficacy, haemocompatible, decreased inflammatory cells, formation of epidermis, and granulation tissue.	[99]
10 mm	14 days	Nanofibers	MgO-NPs	Reduced the expression of inflammatory cytokine interleukin 1-alpha (IL-1α), upregulate collagen production, antimicrobial, and biocompatible.	[72]
1 cm	2 weeks	hydrogels	MgO-NPs	Antibacterial efficacy, no-toxic, promote cell proliferation, and accelerated wound healing.	[100]
7 mm	14 days	Nanofibers	MgO-NPs	Exhibited antibacterial efficacy, cell safety, showed improved hair follicles, blood vessels, mature epidermis, and organized fibroblast.	[101]
10 mm	1 week	Nanofibers	MgO-NPs	Promoted healthy blood vessel and formation, reduced inflammation, of granulation tissue, upregulated M1 to M2 switch of macrophages.	[102]
	12 days	Hydrogels	Mg(OH)_2_-NPs	Haemocompatible, biocompatibility and constrained biofilm formation.	[103]
	72 h	topical gels	MgO-NPs	Promoted rapid clot formation, cell viability of HaCaT cells, high antibacterial activity, and in vitro wound healing.	[104]
	12 h	films	MgO-NPs	Biodegradable and biocompatible high antibacterial activity. Promoted in vitro wound healing	[105]
	48 h	Nanocomposites		Exhibited antibacterial inhibition, non-toxicity, and in vitro wound healing.	[106]
	21 days	Nanofibers	AgNPs & Au-NPs	Reducing inflammatory response, exhibited cell viability and bacterial inhibition, promoted wound healing of Sprague-Dawley rats.	[108]
	24 h	Nanofibers	AgNPs & ZnO-NPs	Exhibited antibacterial efficacy, high fibroblast cells survival rate, antioxidant activity, and in vitro wound scratch healing.	[109]
7 mm	7 days	Composites	AgNPs & ZnO-NPs	Displayed high antimicrobial effect, improved cell viability of L02 cells, tissue regeneration with packed keratinocytes and an organized granulation tissue.	[110]
12 mm	10 days	Xerogels	AgNPs & ZnO-NPs	Showed high bacterial killing effect by generation of ROS, high cell viability, promotes wound reduction, and displayed less inflammatory cells and normal tissue.	[111]
4 cm	10 days	Composite gels	AgNPs & ZnO-NPs	Biocompatible, and promotes raped wound healing.	[112]

## Data Availability

The data are cited and included in this manuscript.
